# Assessment of Attractiveness of Plants as Roosting Sites for the Melon Fly, *Bactrocera cucurbitae*, and Oriental Fruit Fly, *Bactrocera dorsalis*

**DOI:** 10.1673/031.007.5701

**Published:** 2007-11-14

**Authors:** Grant T. McQuate, Roger I. Vargas

**Affiliations:** U.S. Pacific Basin Agricultural Research Center, USDA-ARS, P.O. Box 4459, Hilo, HI

**Keywords:** Suppression, bait spray, crop borders, *Ricinus communis*, *Polyscias guilfoylei*, *Erythrina variegata*, *Psidium guajava*

## Abstract

The use of toxic protein bait sprays to suppress melon fly, *Bactrocera cucurbitae* (Coquillett) (Diptera: Tephritidae), populations typically involves application to vegetation bordering agricultural host areas where the adults seek shelter (“roost”). Although bait spray applications for suppression of oriental fruit fly, *Bactrocera dorsalis* (Hendel), populations have traditionally been applied to the host crop, rather than to crop borders, roosting by oriental fruit flies in borders of some crop species, such as papaya, *Carica papaya* L. (Brassicales: Caricaceae), suggests that bait spray applications to crop borders could also help in suppression of *B. dorsalis* populations. In order to develop improved recommendations for application of bait sprays to border plants for suppression of melon fly and oriental fruit fly populations, the relative attractiveness of a range of plant species, in a vegetative (non-flowering) stage, was tested to wild melon fly and oriental fruit fly populations established in a papaya orchard in Hawaii. A total of 20 plant species were evaluated, divided into four categories: 1) border plants, including corn, *Zea mays* L. (Poales: Poaceae), windbreaks and broad-leaved ornamentals, 7 species; 2) weed plants commonly found in agricultural fields in Hawaii, 6 species; 3) host crop plants, 1 species- zucchini, *Cucurbita pepo* L. (Violales: Curcurbitaceae), and 4) locally grown fruit trees, 6 species. Plants were established in pots and placed in an open field, in clusters encircling protein bait traps, 20 m away from the papaya orchard. Castor bean, *Ricinus communis* L. (Euphorbiales: Euphorbiaceae), panax, *Polyscias guilfoylei* (Bull) Bailey (Apiales: Araliaceae), tiger's claw, *Erythnna variegata* L. (Fabales: Fabaceae), and guava, *Psidium guajava* L. (Myrtales: Myrtaceae) were identified as preferred roosting hosts for the melon fly, and tiger's claw, panax, castor bean, Canada cocklebur, *Xanthium strumarium* L. (Asterales: Asteraceae), Brazilian pepper tree, *Schinus terebinthifolius* Raddi (Sapindales: Anacardiaceae), ti plant, *Cordyline terminate* (L.) Chev.(Liliales: Liliaceae), guava and several *Citrus* spp. were identified as preferred roosting hosts for oriental fruit fly. Guava had not previously been identified as a preferred roosting host for melon fly. Other than for the use of panax as a roosting host, there has previously been little attention to roosting hosts for oriental fruit fly. Establishment of preferred roosting hosts as crop borders may help to improve suppression of both fruit fly species by providing sites for bait spray applications. Further research is needed to assess the use of vegetation bordering other host crops as roosting hosts, especially for oriental fruit fly.

## Introduction

Both male and female melon flies seek shelter (“roost”) in vegetation bordering host areas at night. In the morning, primarily females move into the host crop area for oviposition into the host fruits, while the males mostly remain in the bordering vegetation (Nishida and Bess 1957). Different plant species in Hawaii vary in their attractiveness as roosting sites. Plants shown to be attractive to melon flies for roosting include crop plants such as corn, *Zea mays* L. (Nishida and Bess 1957; McQuate et al. 2003), guava, *Psidium guajava* L., and citrus varieties (Kazi 1976); border (windbreak) plants such as tiger's claw, *Erythrina tahitensis* Nadeaud (Stark 1995); and weeds such as castor bean, *Ridnus communis* L., spiny amaranth, *Amaranthus spinosus* L., and fuzzy rattlepod, *Crotalaria incana* L. (Nishida and Bess 1957; Kazi 1976).

Based on these behavioral observations, an effective control method was developed for melon fly wherein a bait, with an added toxicant, was sprayed along bordering attractive vegetation. The bait reduces the proportion of crop or land area that must be covered with spray droplets compared with application of pesticide alone in conventional sprays (Prokopy et al. 1992). Enzymatic protein hydrolysates became the baits of choice because of their attractiveness as food sources, while malathion became the organophosphate insecticide of choice because of its low mammalian toxicity, low price, and low levels of fruit fly resistance (Steiner 1952; Steiner et al. 1961; Roessler 1989). However, overuse of organophosphate insecticides has been implicated in secondary pest outbreaks, negative effects on beneficial insects, environmental contamination and adverse effects on human health (Troetschler 1983; Hoy and Dahlsten 1984; Marty et al. 1994). Consequently, more environmentally friendly replacements for these compounds have been sought (McQuate et al. 1999 and 2005a,b; Peck and McQuate 2000; Vargas et al. 2002; Barry et al. 2003; Prokopy et al. 2003; Stark et al. 2004).

If plants bordering melon host cropping areas are not attractive to melon flies they are not a good site for application of bait sprays. In these cases, attractive plants such as corn or castor bean could be planted along crop borders to serve as a point of localization for the bait spray (Nishida and Bess 1957; Kazi 1976). At present in Hawaii, borders of cropping areas attractive to the melon fly may have plants planted for reasons other than melon fly control (e.g., windbreaks), or may have an array of weedy plants of varying attractiveness to melon fly, or may include other perennial or annual crops. Weeds of various levels of attractiveness may also develop within the crop. A fruit fly bait recently registered for use in Hawaii, GF-120 Fruit Fly Bait (Dow AgroSciences, www.dowagro.com) has a maximum application rate of 4.0 liters/ha. Because it can be difficult to distribute this low quantity as a ground spray, a study was initiated to assess the attractiveness of diverse vegetation found in two melon fly host cropping areas in Hawaii (Kamuela, Hawaii and Kula, Maui) in order to improve recommendations for planting of crop borders for melon fly control and for establishing priorities for placement of limited spray volumes. Because the site selected for the study (see below) also had a well-established oriental fruit fly population, the use of vegetation in crop borders by oriental fruit fly could also be assessed.

The behavior of roosting and feeding in border areas is not as well documented for oriental fruit fly as it has been for melon fly. Stark et al. (1994) reported that oriental fruit flies spent the entire day in and around guava trees (including night-time roosting), so it would not be expected to make use of roosting hosts bordering a guava orchard. In contrast, in papaya (*Carica papaya* L.) orchards, Stark (1995) noted that oriental fruit flies left papaya trees around dusk (Stark 1995) and roosted in windbreaks bordering the orchard, especially panax (*Polyscias guilfoylei* [Bull] Bailey). Stark suggested that the roosting behavior of oriental fruit fly on panax could be used for control purposes through insecticide applications on the panax plants. He further noted that oriental fruit flies may roost in other plant species at other sites. Since these observations were made, no further work has been done in identifying roosting hosts for oriental fruit fly and in testing the effectiveness of border applied bait spray applications for control of oriental fruit fly populations.

Plants that could potentially be grown in crop borders as windbreaks, weed species found in melon fly host cropping areas, fruit trees which may be found in melon fly host cropping areas and one melon fly host crop were established in pots and brought to a site known to have well established populations of both melon fly and oriental fruit fly and used in tests to compare their relative attractiveness to these two fly species. Papaya orchards provide an excellent site to test for relative attractiveness of different plant species because field sanitation is generally not practiced in commercial papaya orchards in Hawaii. Fruits at the “color break” stage are typically harvested for sales while riper fruits either fall off later, or are knocked to the ground, and are left to rot on the ground. These ground fruit are readily infested by both melon flies and oriental fruit flies and serve as a reservoir of resident populations of both melon fly and oriental fruit fly (Liquido 1991 and 1993). By conducting our field trials at a site with both melon fly and oriental fruit fly populations, we were able to assess border plant use by wild flies of both fruit fly species.

**Figure 1.**
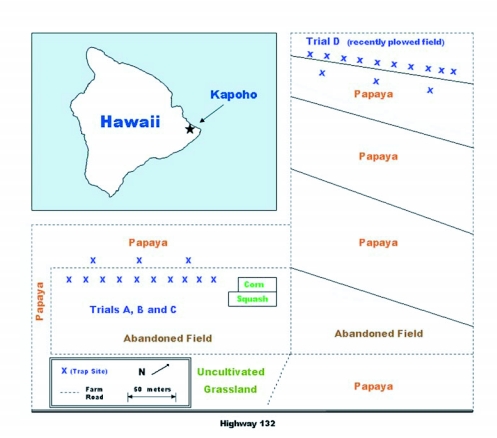
Locations of fields in which potted plants were placed relative to papaya orchards.

**Table 1.**
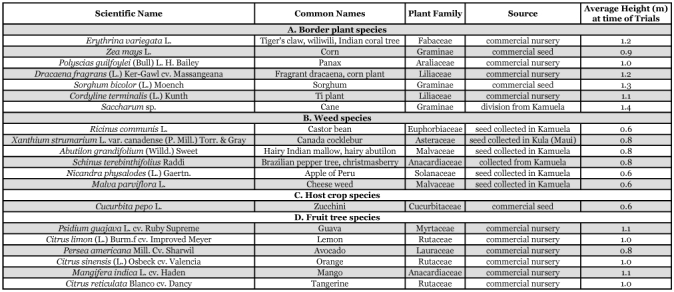
List of plant species tested for attractiveness to melon fly, *Bactrocera cucurbitae*, and oriental fruit fly, *Bactrocera dorsalis*.

**Table 2.**
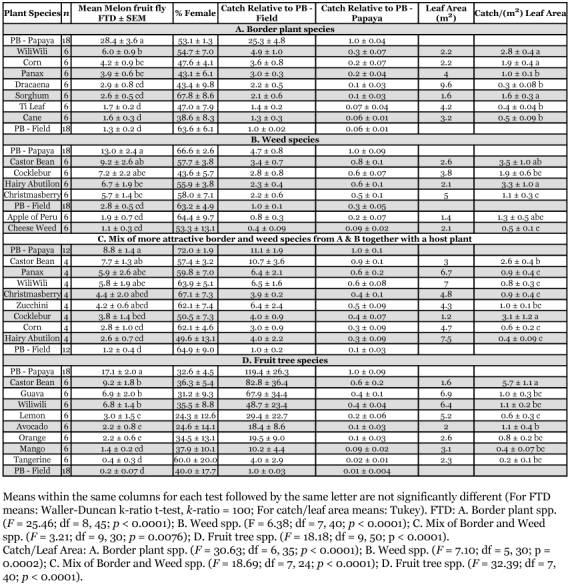
Average melon fly catch (flies/trap/day [FTD] ± SEM) in protein bait traps. Also listed, for each trial, are catches in protein bait traps in the field, not associated with any plant species (PB - Field) and in protein bait traps in the bordering papaya orchard (PB - Papaya).

## Materials and Methods

### Study site

Field trials were conducted in the environment of papaya orchards in Kapoho, Hawaii (see Figure 1). Papaya, a tropical fruit, is produced year-round in orchards at this site. All orchards adjacent to the trials were producing ripe fruits and had varying levels of ground fruits that supported large wild populations of both melon fly and oriental fruit fly.

### Plant species tested

The following separate trials were conducted: (A) Border Plant Trial (conducted 27 Feb. - 23 March, 2002). Plants currently in use as border plants, or of potential value as border plants, of cucurbit growing areas of Hawaii were identified, obtained as either seeds or plants and grown and/or maintained in pots. These plants included corn (*Zea mays* L.) (Poales: Poaceae), a plant previously identified as attractive to tephritid fruit flies (Nishida and Bess 1957; McQuate et al. 2003) as well as several plant species used in Hawaii as windbreaks and several broad-leaved ornamentals; (B) Weed Species Trial (conducted 28 March — 21 April, 2002). Seeds of weed species present in cucurbit growing areas of Hawaii were collected and sown, with plants established in pots; (C) Border, Weed and Host Trial (conducted 8–24 May, 2002). The three border plant species most attractive to melon fly (two of these three species were included in the top 3 most attractive plants for oriental fruit fly, also [Trial A results]), the four weed species most attractive to melon fly (which were also the 4 most attractive weed species for oriental fruit fly [Trial B results]), and a common commercially grown zucchini, *Cucurbitapepo L*. (Violales: Curcurbitaceae) were all established in pots. All male and female flowers were routinely picked from the zucchini plants in order to have attraction to the zucchini plants based on attraction to foliage (attractiveness as a roosting plant) and not to fruits (attractiveness as a host plant); (D) Fruit Tree Trial (conducted 15 Oct. — 4 Nov., 2002). Fruit tree species previously reported to be attractive to melon flies, or found to be sites of higher melon fly catch in cue-lure baited traps of the melon fly suppression program, were obtained from a local fruit tree nursery and maintained in pots. Additionally, the weed species in Trial C that was most attractive to both melon fly and oriental fruit fly (castor bean) and one of the most attractive border plant species from this trial (tiger's claw) were also included in the Fruit Tree Trial to provide comparison with results from the earlier trials. Plant species included in the four trials are listed, by plant type, in Table 1. Tables 2 and 3 list the specific plant species used in each trial.

**Table 3.**
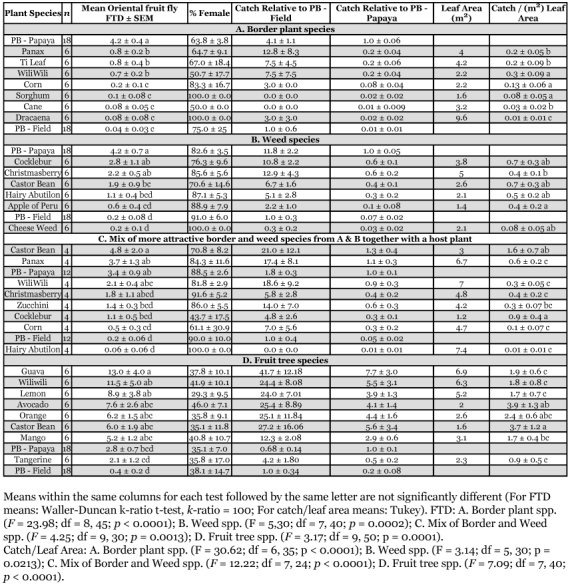
Average oriental fruit fly catch (flies/trap/day [FTD] ± SEM) in protein bait traps ± laced in A.) Different border plant species; B.) Different weed species; C.) Mix of more attractive border plant and weed species from A and B together with a host plant; and D) Fruit tree species. Also listed, for each trial, are catches in protein bait traps in the field, not associated with any plant species (PB - Field) and in protein bait traps in the bordering papaya orchard (PB — Papaya).

**Figure 2.**
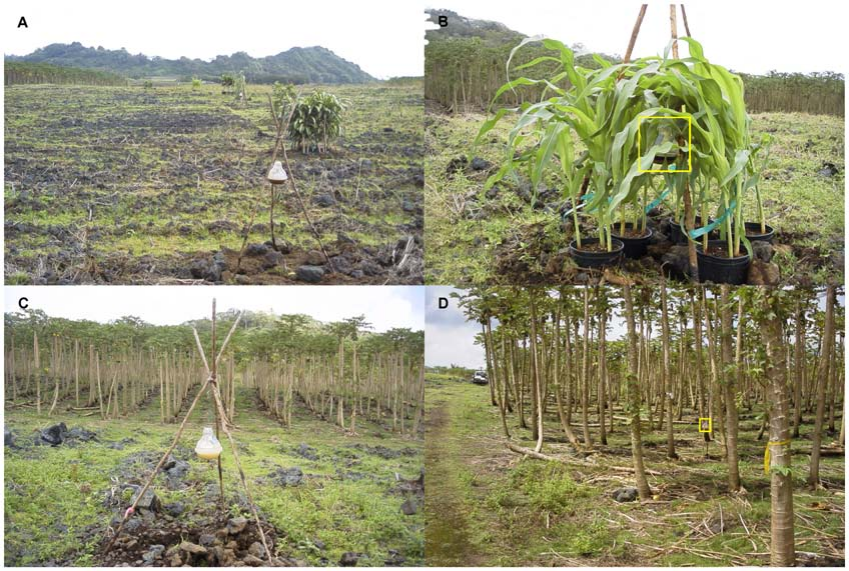
A. Overview of trial set-up with papaya orchard to the left and protein bait traps, placed both with and without (Control: “PB-Field”) association to clusters of pots of test plants arranged 20 m from the border of the papaya orchard; B. Close-up of plant cluster (here, corn) showing central positioning of protein bait trap (inside yellow square); C. Control (without association with potted plants) protein bait trap, in open field 20 m from the border of the papaya orchard; and D. Protein bait trap (inside yellow square; Control: “PB-Papaya”) hung from “tripod” positioned between papayatreesin from the border.

### Bioassay

Within a trial, clusters of 5 pots of plants of each species tested were set out 20 m from the edge of a papaya orchard in Kapoho, Hawaii (see Figure 2). The field in which the pots were placed was fallow. For trials A and B, larger weeds between the plant clusters and the papaya orchard were pulled by hand to minimize opportunity for fruit flies to roost on plants other than the test species presented. For trial C, Roundup Ultra (Monsanto, www.monsanto.com) was applied, one week before the start of the test, to the weeds between the papaya orchard and the test plants and for about 4 m beyond the test plants. The distance from the papaya orchard chosen had been found to yield fly response to the plant cluster but reduced direct response to the protein bait trap (GTM, unpublished data). A plastic dome trap with a clear bottom (Biosys, Inc., Palo Alto, CA, U.S.A.) baited with a protein bait solution (8% Solulys, [Roquette America, Inc., roquette.com]; 4% Borax; 88% water) was hung within each cluster of 5 pots of plants. Three similar protein bait traps were hung without association to any plants at three separate points along the row of plant clusters, two near the ends of the row and one near the middle of the row. These traps provided a control for attraction to the bait only. Plant, and protein bait trap alone, clusters were about 10m apart within the row. Position within the row was determined randomly. Protein bait traps were also placed between the second and third tree in from the edge of the papaya orchard to monitor the source tephritid fruit fly population levels. All protein bait traps were serviced every 2 days with location of all plant clusters and protein bait only traps moved to a new ‘random’ orientation every 4 days. Protein bait traps were “topped-off” with fresh protein bait solution at each service and totally replaced after 12 days (three 4-day cycles). For single class trials (e.g., border plants, weeds, or fruit trees) six 4-day cycles were completed for each set of plants. For combination trials (e.g., Trial C: border plants, weeds and host), only four 4-day cycles were completed.

### Calculation of leaf areas of test plant species

In order to permit standardization of catch by leaf area, total leaf area was estimated for each plant cluster, the technique used varying depending on the size and shape of the leaves. This was done because equivalent leaf areas could not readily be presented for all species tested. For plants with larger leaves, but having widths narrower than 15.0 cm [corn, sorghum, tiger's claw, sugar cane, fragrant dracaena (hereafter referred to as dracaena), Canada cocklebur (hereafter referred to as cocklebur), avocado, guava, and mango] leaf area was estimated using a CI-203 portable laser area meter (CID, Inc., www.cid-inc.com). For plants with leaves wider than 15.0 cm (castor bean, ti plant, hairy Indian mallow, *Abutilon grandifolium* (Willd.) Sweet [Malvales: Malvaceae], and zucchini), leaf area was approximated by the sum of the products, from each leaf, of leaf length (*L*), maximum leaf width (*W*), and a correction factor, similar to a leaf area estimation procedure used with corn (Zhang and Brandie 1997). For plants with many small leaves (panax, Apple of Peru, cheese weed, Brazilian pepper tree, tangerine, lemon, and orange) a gravimetric method was used.

### Statistical analyses

Catch for each plant species tested was replicated in time, but not in space as tests for plant species were replicated in subsequent, re-randomized runs, but there were no replicates of any plant species at any one time. The two 2-day trap catches at each plant associated cluster for a given randomization were combined for each cycle, effectively providing an average catch response for each cycle. The two two-day catches for each protein bait only trap were also combined and then the combined totals were averaged over the three protein bait-only traps set out in line with the potted plants in the field next to the papaya orchard and over the three protein bait-only traps in the papaya field to give average catches for each cycle. These results were replicated over a total of 6 cycles (Trials A, B, & D) or 4 cycles (Trial C). Catch was converted to flies per trap per day before data transformation and analysis. Catch for plant species relative to the catch at the field protein bait traps not associated with plants (PB-Field) and relative to the protein bait catch in the papaya orchard (PB-Papaya) were calculated as the average over all cycles within a trial. Combined trap catch was square root transformed [sq rt (x + 0.5)] (Sokal and Rohlf, 1981) and subjected to analysis of variance (ANOVA), with Waller-Duncan K-ratio T Test for means separation (SAS Institute 1998). Square root transformed catch per trap per day divided by leaf area was also analyzed by ANOVA, with Tukey HSD for means separation (JMP 2002). Percentage female catch was arcsine transformed [arcsin (sq rt (%/100))] (Sokal and Rohlf, 1981) and subjected to analysis of variance (ANOVA) (JMP 2002). Tables summarizing bioassay results present untransformed trap catch results together with statistical results based on transformed values.

## Results

There were significant differences in trap catch among treatments for both melon fly and oriental fruit fly in all 4 trials. Average trap catch results, for all four trials, together with ANOVA results, are presented in Table 2 (melon fly catch) and Table 3 (oriental fruit fly catch). Each table also presents the percentage female catch, the catch in plant clusters relative to isolated protein bait traps in the field and to protein bait traps in the papaya orchard, the estimated total leaf area of each plant cluster and the average catch per m2 of leaf area for each plant cluster. In all four trials, for both fly species, there was no significant difference in percentage female catch among treatments. Aspects where there were significant differences among treatments in trap catch are presented below, by fly species, for each trial.

## Melon fly

### Border plant species trial

Traps associated with tiger's claw, corn and panax had higher melon fly catch than traps placed near other plant species, though the catch associated with corn and panax was not significantly greater than the catch associated with dracaena or sorghum (Table 2a). Catch associated with tiger's claw, corn and panax exceeded catch in the field protein bait-only traps by about 3–5 times, but was only about 20 to 25% of the trap catch in the papaya orchard. When trap catch was adjusted by leaf area, tiger's claw and corn remained the top two attractive plants, while sorghum replaced panax as the third most attractive plant. Adjusted trap catch for these three plant species was significantly greater than catch for any of the other species tested.

### Weed species trial

Traps associated with castor bean captured the highest number of melon flies, but not significantly higher than captures in traps placed near cocklebur, hairy Indian mallow, and Brazilian pepper tree (Table 2b). Catch associated with these 4 species exceeded catch in the field protein bait-only traps by about 2–3 times, but was only about 50 to 75% of the trap catch in the papaya orchard. Following adjustment of trap catch by leaf area, catch per m^2^ remained highest at the same top three species (castor bean, cocklebur and hairy Indian mallow) with catch per m^2^ leaf area numerically greatest for castor bean, but catch per m^2^ associated with hairy Indian mallow became significantly higher than catch associated with cocklebur.

### Mixed border, weed and host species trial

Among traps associated with plant species, melon fly catch was highest in traps placed near castor bean, panax, tiger's claw, Brazilian pepper tree and zucchini (Table 2c). Trap captures associated with these plant species were less than, but not significantly different from, trap captures in the papaya orchard. Catch associated with these 5 species exceeded catch in the field protein bait-only traps by about 6–10 times, but was only about 50 to 90% of the trap catch in the papaya orchard. Following adjustment of trap catch by leaf area, traps associated with cocklebur had significantly higher catch than traps associated with any other plant species. Traps associated with castor bean had the second highest trap catch per m^2^ leaf area, but this catch was not significantly different than catch associated with zucchini.

### Fruit tree species trial

Traps placed near castor bean, guava, and tiger's claw had significantly higher melon fly catch than traps associated with any other roosting host (Table 2d). The next most attractive hosts were lemon, avocado, and orange. Catch associated with the 3 most attractive species exceeded catch in thefield protein bait-only traps by about 35–48 times, but was only about 40 to 54% of the trap catch in the papaya orchard. Catch per unit leaf area was significantly higher for castor bean than for any other roosting host.

## Oriental fruit fly

### Border plant species trial

Oriental fruit fly catch in traps placed near panax, ti plant, and tiger's claw was significantly higher than catch in traps placed near any other roosting host (Table 3a). Catch associated with these 3 most attractive species exceeded catch in the field protein bait-only traps by about 20 times, but was only about 16 to 20% of the trap catch in the papaya orchard. Captures near tiger's claw were significantly higher than captures near panax or ti plant based on catch per unit leaf area.

### Weed species trial

Oriental fruit fly captures in traps placed near cocklebur and Brazilian pepper tree were higher than captures associated with other hosts, but captures were not significantly greater than captures associated with castor bean or hairy Indian mallow (Table 3b). Catch per unit leaf area was similar among the different roosting plant species.

### Mixed border, weed and host species trial

Oriental fruit fly captures in traps placed near castor bean were greater than captures in traps placed near other hosts, but not significantly greater than captures in traps placed near panax, tiger's claw or Brazilian pepper tree (Table 3c). Captures in traps placed near castor bean and near panax were numerically greater (but not significantly) than captures in the papaya orchard. Catch per unit leaf area was significantly higher for cocklebur than for any other roosting host except castor bean.

### Fruit tree species trial

Oriental fruit fly captures in traps placed near guava were greater than captures associated with other hosts, but were not significantly greater than captures associated with tiger's claw, lemon, avocado, orange, castor bean, or mango (Table 3d). Catch associated with guava was, however, significantly greater than catch in the papaya orchard. Catch per unit leaf area was highest for avocado, castor bean, and orange, with catch associated with castor bean significantly greater than catch associated with all of the other roosting hosts.

## Discussion

For melon fly, these results provide further support for some of the roosting hosts identified in earlier studies (e.g., castor bean and tiger's claw), provide variable results for the value of corn as a roosting host and identify several additional plant species which can be good roosting hosts (e.g., guava and cocklebur). In all three trials in which castor bean was included, catch associated with castor bean exceeded catch with any other plant species tested. No other plant had such consistently superior results. Although castor bean is a weed species, it can be maintained as a trap crop along the border of a melon host crop, providing a focal point for bait sprays. When used as such, it is best to cultivate it in patches, rather than as an extended continuous border, to minimize the volume of bait spray required to treat the border areas. This technique has been used effectively on the border of a zucchini field on Maui.

The windbreaks (panax and tiger's claw) were found to be generally of equal attractiveness to melon flies, though an earlier study (Stark 1995) had found that melon fly preferentially roosted in tiger's claw plants. Clearly, both species are attractive, but preference between the two species may vary with location.

Corn, which had previously been identified as an attractive plant for melon flies (Nishida and Bess 1957; McQuate et al. 2003), performed well in the first trial, but ranked toward the bottom of the mixed border and weed species trial. Similar variability of use had also been noted with released sterile male melon flies (Peck et al. 2005). Corn is, however, one roosting plant species for which information on roosting at different phenology stages has been documented. It has been noted that both melon fly and oriental fruit fly may show increased population levels in corn at the time of, and subsequent to, flowering and pollen shed (McQuate et al. 2003).

Additional favorable melon fly roosting hosts identified in this paper include cocklebur and guava. Nishida and Bess (1957) had reported finding melon fly roosting in cocklebur fairly commonly, but found that the level of roosting in guava was marginal compared to other roosting hosts. In our study, guava proved to be a good melon fly roosting host, with catch in traps hung near guava not significantly different than the best roosting hosts identified (castor bean and tiger's claw). Sorghum and cane, planted as crop borders by some farmers in Hawaii as a windbreak and a focal point for bait sprays, were significantly less attractive than tiger's claw. However, once adjusted for leaf area, catch associated with sorghum, though still less, was not significantly different than catch associated with tiger's claw. Clearly leaf area is an important issue. Prokopy et al. (2004) reported that they found bait spray application to narrow sorghum borders to be less effective for melon fly control than application to broader sorghum borders. Increasing width or density of other border plants tested here may similarly improve their effectiveness as sites for bait spray application.

The catch associated with nonflowering or nonfruiting zucchini foliage is interesting because the typical understanding of melon fly behavior is that immature males and females roost and forage in areas bordering host crops and fertile females enter the host areas for purposes of oviposition (Nishida and Bess 1957). Here, however, catch (both male and female) associated with nonflowering/nonfruiting zucchini was not significantly less than catch associated with known roosting hosts. The catch of flies associated with zucchini suggests that melon flies find zucchini foliage to be attractive for roosting, so may less readily depart zucchini crops for crop borders, which may also be true for other broad-leaved cucurbit crops.

It should be noted that our trials were all based on trap captures, whereas Nishida and Bess (1957) also made use of sweep net captures. The use of protein-baited traps gives a good record of where flies will respond to protein baits, but, unless deployed over short intervals, fail to show the exact time of day that the flies were at a given site. Sweep nets, on the other hand, can provide documentation of the flies present at a specific time, without regards to how readily they would respond to a protein bait. However, if only a few flies are present, protein baits permit accumulation over time to document fly presence, whereas multiple sweepings would be required to get adequate captures to provide good fly location data. Both techniques are useful, if not complementary. We, however, chose not to use sweep nets because of perceived difficulty in catching flies located in the center of the plant clusters, concern for damage of limited numbers of plants available for each species, and low fly numbers in the less preferred roosting hosts.

Other than identifying panax as a roosting host, little attention has been given to roosting hosts for oriental fruit fly. This study has identified a number of plant species which can be used by oriental fruit flies as roosting hosts, including castor bean, Brazilian pepper tree, cocklebur, and ti plant, as well as a number of different fruit trees during nonflowering/nonfruiting stages.

**Figures 3.**
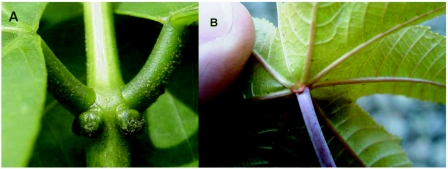
Extrafloral nectaries in A. Tiger's claw; and B. Castor Bean.

In regards to panax, this study has found, as had Stark (1995), that this plant is attractive as a roosting host for the oriental fruit fly. In both trials of this study, where both panax and tiger's claw were tested, catch associated with panax exceeded that associated with tiger's claw, but was not significantly greater in either trial. Although leaves and extracts of panax are known to be attractive to female oriental fruit flies (Jang 1997), no research has yet been directed to the source of the attractiveness of tiger's claw leaves. It should be noted that tiger's claw leaves have extrafloral nectaries (Figure 3a), comparable to those found in castor bean (Figure 3b) and Nishida (1958) documented that oriental fruit flies (as well as melon flies) fed on nectar from extra-floral nectaries in castor beans. The value of tiger's claw as a roosting host in Hawaii has recently been adversely impacted by the invasion (subsequent to our studies) of a new pest species, the Erythrina gall wasp, *Quadrastichus erythrinae* Kim (Hymenoptera: Eulophidae) (Heu et al. 2006). This wasp has also adversely affected *Erythnna* spp. in Taiwan (Yang et al. 2004), Singapore, Mauritius and Reunion (Kim et al. 2004), and India (Faizal et al. 2006). This wasp inserts eggs into young leaf and stem tissues. The resulting larvae induce the formation of galls in leaflets and petioles. Severe infestations are reported to cause defoliation and death of trees (Yang et al. 2004).

The results from the fruit tree trial provide further insight into use of orchard borders by oriental fruit fly. Stark et al. (1994) concluded that oriental fruit fly, as well as the parasitoid species associated with it, remain in a guava orchard throughout the day and night. In the present study, catch of oriental fruit flies was significantly greater in an adjacent small cluster of vegetative guava plants than in the papaya orchard. Although catch of oriental fruit flies might have been greater in the papaya orchard if the traps had been placed closer to the fruit and foliage, among all plants tested here it was only in guava that catch significantly exceeded catch in the papaya orchard. For all five other vegetative fruit trees (lemon, avocado, orange, mango and tangerine) the catch was not significantly different than the catch associated with guava trees, and the average catch for four of these (all but tangerine) numerically exceeded the catch in the papaya orchard, but was not significantly greater. These results are suggestive that oriental fruit flies may similarly form resident populations in orchards of these other species, as in guava. However, it should be noted that the guava orchard used in the Stark et al. (1994) study did not have tiger's claw or panax borders. It is possible that, if a guava orchard (or other oriental fruit fly host orchard) had tiger's claw or panax borders, a portion of the population may use these as roosting sites, and not just remain in the orchard. Further research is yet needed to assess the use of borders by oriental fruit fly under varied orchard/border scenarios. Knowledge of distribution of the flies is important for decisions as to how to apply bait sprays. Although cover sprays of a protein bait + toxicant have been typically employed for suppression of oriental fruit fly (e.g., McQuate et al. 1999), it's clear that the relative importance of border applications may vary from host to host and with different orchard borders.

Identification of attractive nonhosts provides a basis for one means of improving the effectiveness of bait sprays for melon fly control as suggested by Prokopyet al. (2003). This issue is also true for oriental fruit fly, and perhaps for other tephritid fruit fly species as well. Although attractive plants have been identified in this paper, the sexual maturity or protein status of the attracted flies was not determined, so we cannot state that these plants are attractive to both protein-satiated and protein-hungry females as recommended by Prokopy et al. (2003). Additionally, all plants tested were in a vegetative stage, so it is not known how flowering or fruiting might affect attractiveness of these plant species as roosting sites. However, from the perspective that flowering and fruiting could provide additional adult food sources, one would anticipate that flowering and fruiting might enhance the attractiveness of the plants tested here as roosting sites, as shown previously for corn (McQuate et al. 2003). Further research is needed to address these questions and to better document the relative attractiveness of host species and nonhost plant species used as roosting sites. As our understanding of roosting behavior improves, it will be easier to establish priorities for species selection for crop borders as well as to improve targeting of bait sprays to optimize population suppression of these tephritid fruit fly species.

## **Abbreviations**

PB -: protein bait
FTD -: flies per trap per day
